# Knee osteoarthritis and time-to all-cause mortality in six community-based cohorts: an international meta-analysis of individual participant-level data

**DOI:** 10.1007/s40520-020-01762-2

**Published:** 2021-02-15

**Authors:** Kirsten M. Leyland, Lucy S. Gates, Maria T. Sanchez-Santos, Michael C. Nevitt, David Felson, Graeme Jones, Joanne M. Jordan, Andrew Judge, Dani Prieto-Alhambra, Noriko Yoshimura, Julia L. Newton, Leigh F. Callahan, Cyrus Cooper, Mark E. Batt, Jianhao Lin, Qiang Liu, Rebecca J. Cleveland, Gary S. Collins, Nigel K. Arden, The PCCOA Steering Committee, Lyn March, Gillian Hawker, Philip Conaghan, Virginia Byers Kraus, Ali Guermazi, David Hunter, Jeffrey N. Katz, Tim McAlindon, Tuhina Neogi, Lee Simon, Marita Cross, Lauren King

**Affiliations:** 1 MRC Integrated Epidemiology Unit, Population Health Sciences, University of Bristol, Bristol, UK; 2 Nuffield Department of Orthopaedics, Rheumatology and Musculoskeletal Sciences, University of Oxford, Oxford, UK; 3 Centre for Sport, Exercise and Osteoarthritis Research Versus Arthritis, University of Southampton, Southampton, UK; 4 Centre for Statistics in Medicine, Nuffield Department of Orthopaedics, Rheumatology and Musculoskeletal Sciences, University of Oxford, Oxford, UK; 5 Department of Epidemiology and Biostatistics, University of California, San Francisco, San Francisco, CA USA; 6 Boston University School of Medicine, Boston, MA USA; 7 Menzies Institute for Medical Research, University of Tasmania, Hobart, Australia; 8 Department of Medicine, University of North Carolina School of Medicine, Chapel Hill, NC USA; 9 Musculoskeletal Research Unit, Translational Health Sciences, Bristol Medical School, University of Bristol, Bristol, UK; 10 Department of Preventive Medicine for Locomotive Organ Disorders, 22nd Century Medical and Research Center, The University of Tokyo, Tokyo, Japan; 11 MRC Lifecourse Epidemiology Unit, University of Southampton, Southampton General Hospital, Southampton, UK; 12 Centre for Sport, Exercise and Osteoarthritis Research Versus Arthritis, Nottingham University Hospitals, Nottingham, UK; 13 Peking University People’s Hospital, Arthritis Clinic and Research Centre, Beijing, China

**Keywords:** Osteoarthritis, Knee, Mortality, Meta-analysis

## Abstract

**Background:**

Osteoarthritis (OA) is a chronic joint disease, with increasing global burden of disability and healthcare utilisation. Recent meta-analyses have shown a range of effects of OA on mortality, reflecting different OA definitions and study methods. We seek to overcome limitations introduced when using aggregate results by gathering individual participant-level data (IPD) from international observational studies and standardising methods to determine the association of knee OA with mortality in the general population.

**Methods:**

Seven community-based cohorts were identified containing knee OA-related pain, radiographs, and time-to-mortality, six of which were available for analysis. A two-stage IPD meta-analysis framework was applied: (1) Cox proportional hazard models assessed time-to-mortality of participants with radiographic OA (ROA), OA-related pain (POA), and a combination of pain and ROA (PROA) against pain and ROA-free participants; (2) hazard ratios (HR) were then pooled using the Hartung–Knapp modification for random-effects meta-analysis.

**Findings:**

10,723 participants in six cohorts from four countries were included in the analyses. Multivariable models (adjusting for age, sex, race, BMI, smoking, alcohol consumption, cardiovascular disease, and diabetes) showed a pooled HR, compared to pain and ROA-free participants, of 1.03 (0.83, 1.28) for ROA, 1.35 (1.12, 1.63) for POA, and 1.37 (1.22, 1.54) for PROA.

**Discussion:**

Participants with POA or PROA had a 35–37% increased association with reduced time-to-mortality, independent of confounders. ROA showed no association with mortality, suggesting that OA-related knee pain may be driving the association with time-to-mortality.

**Funding:**

Versus Arthritis Centre for Sport, Exercise and Osteoarthritis and Osteoarthritis Research Society International.

## Introduction

The prevalence of musculoskeletal disorders (not including back pain) was ranked 19th for men and 20th for women in the 2017 Global Burden of Disease study. Knee OA made up 20% of this musculoskeletal burden. In terms of living with disability, musculoskeletal disorders ranked 10th and 11th for men and women, respectively [1]. The lifetime risk of knee osteoarthritis is estimated to be 45% [2], and the prevalence of knee OA is expected to rise in accordance with the increase in the ageing population and obesity epidemic in many parts of the world.

OA is a common debilitating joint disease, frequently associated with joint pain, functional limitation, and decreased quality of life [3]. It most commonly affects the knees, hips, hands, facet joints, and feet [4], with knee and hip OA causing the greatest burden to the population, as pain and stiffness in these large weight-bearing joints often lead to significant physical dysfunction such as knee muscle weakness and limited flexion [5].

Since 2008, ten studies and three meta-analyses have reported the association between knee OA and mortality, with only a handful of studies before this time [6–9]. Varied findings of both positive and negative associations have made it difficult to draw conclusions regarding the effects of OA on mortality [8, 10–16].

This variation in findings reflects differences in populations studied (clinical or general), the diagnostic methods used to define OA, statistical methodology used, and the use or inclusion of important confounders in each study. Traditional meta-analyses are valuable and efficient in terms of time and resources required, but do have several limitations, which have been widely recognised [17–19] including reliance by necessity on published data increasing the potential for publication bias as negative studies difficult to publish. Aggregate data are often not available, poorly reported, derived, and presented differently across studies (for example, odds ratio versus relative risk), and most studies vary in their definitions of exposures, confounders and outcomes [20].

Individual patient-level (IPD) meta-analysis utilises original raw data from cohorts and uses standardised statistical methods to analyse and produce pooled estimates [21]. IPD meta-analysis, although time-consuming and resource intensive, does not depend on previously published data, allows for a standardised definition of important variables and can be analysed using the same statistical approach. Within the current study, key measures of OA and relevant confounders are harmonised (based on expert consensus) [22], and consistent methods of analyses are used between cohorts to provide a more generalizable estimate of the association between OA and premature mortality in the general population.

This study seeks to overcome the limitations introduced when using aggregated results by gathering and analysing individual participant-level data from multiple international observational osteoarthritis cohort studies to describe the association between knee osteoarthritis and time-to all-cause-mortality.

## Methods

### Study design

This study was designed to assess the relationship between knee osteoarthritis and time-to all-cause-mortality in multiple, prospective, longitudinal, community-based cohort studies from around the world. Subjects were stratified by the presence or absence of osteoarthritis at baseline, and time-to-mortality was compared between groups. Pooled estimates were produced using a two-stage individual participant-level meta-analysis framework consisting of two discrete steps: (1) analysing the individual cohorts separately; and (2) applying traditional meta-analysis methods to produce a pooled effect size [21].

A two-stage analysis can more easily handle cohort-specific characteristics such as heterogeneous populations, different risk relationships (such as direction and shape), and the effect of confounders, and can more overtly handle both sporadic and systematic missing data, unlike a one-stage analysis (i.e., pooling all data) [23]. A two-stage analysis allows for consistently defining the primary risk factors, outcome variables, adjusting for the same confounders, and using consistent statistical methods before producing a single pooled effect size. Unlike a traditional meta-analysis, it also allows for the inclusion of previously unpublished data.

### Cohort and participant inclusion/exclusion criteria

Due to the type of data required (detailed pain and radiographic data), and the desire to use cohorts, including those which had not been previously published on the OA/mortality relationship, we identified cohorts using two sources: (1) published literature of cohort studies on knee osteoarthritis and mortality; and (2) contacting principal investigators of longitudinal osteoarthritis cohorts to see whether mortality data had been collected. We did not conduct a traditional systematic review, and as evidenced by the three recent systematic reviews and meta-analyses, several of the cohorts we have included in our study would not have been identified [7–9].

The inclusion criteria for cohorts were: (1) OA-related knee pain and knee radiographic data available at baseline for both OA and non-OA subjects; (2) time-to-mortality follow-up data for all participants; and (3) recruitment from the community (i.e., not identified through clinics, hospitals, or healthcare professionals). Exclusion criteria were: (1) cohorts where raw data could not be released for analysis; and (2) data not available for both OA and non-OA subjects. Cohorts were not selected with regard to previously published data on the relationship between OA and mortality.

We identified 40 cohorts via the two previously described sources as potentially having knee osteoarthritis data from the general population. Eighteen were excluded due to being a non-observational cohort or non-community-based or a case–control study. Thirteen lacked the appropriate knee X-ray or pain data at baseline after more detailed investigation, and two lacked available mortality or time-to-death data. Seven potentially eligible cohorts were identified, one of which had data access limitations, leaving six cohort studies available for analysis (see flow chart, Appendix 1). The six cohorts included were: three US community-based cohorts (Framingham and Johnston County Osteoarthritis Project) [24, 25], one of which was enhanced for OA risk factors [Multicentre Osteoarthritis Study (MOST)] [26]; one community-based cohort from the United Kingdom (Chingford) [27]; one Chinese community-based cohort (Wuchuan) [28]; and one Australian community-based cohort [The Tasmanian Older Adult Cohort (TasOAC)] [29]. All cohorts provided data for all participants except Framingham which provided a random sample of 80%.

Key differences between cohorts (Appendix 2) are the year of baseline visit, length of follow-up, the baseline age of participants, and the lack of side-specific pain in a single cohort. Participants were included in the analysis if they were over 45 years of age, did not have evidence of rheumatoid arthritis, and had mortality data available. After initial data checks, subjects above the age of 80 were also excluded due to the extremely small numbers available (Appendix 2).

### Data collection process

IPD was requested from the principle investigators of any identified cohort after submitting an analysis plan for their team to review. Principle investigators were also contacted directly in cases where data had never been previously released to outside research teams.

A subset of the full data containing only the pre-specified exposures, outcomes, and confounders was requested, transferred via encrypted online servers, and stored and managed centrally by the Oxford research team. A open email dialogue was maintained with principle investigators and key researchers from each cohort throughout the process of data acquisition, harmonisation, and analysis to ensure consistency between cohorts.

### Primary risk factor: knee osteoarthritis

Due to the importance of using a consistent definition of osteoarthritis to avoid misclassification, we gained expert opinion on methods to harmonise knee osteoarthritis variables in prospective OA cohort studies, and all OA criteria used in this analysis were defined following a process of expert consultation, analysis, and agreement [22]. The key output of this meeting supported the use of both a binary self-reported pain question and the presence of radiographic OA to define knee OA in the general population. Thus, knee pain was defined using either an NHANES-type question (i.e., ‘have you had pain for at least a month in the last month in your joint’), or a similar alternative pain question if an NHANES-type question had not been used to assess pain [30, 31]. In cases where only WOMAC was available a threshold of 3 was used on the WOMAC pain subscale, this threshold was determined by the previous expert consensus and external validity study [22]. Radiographic OA was defined using the Kellgren and Lawrence (K/L) scoring method, grade 2, or above, and alternatively, an equivalent combination of radiographic features (osteophytes and joint space narrowing) from other validated scoring methods (such as the OARSI atlas) [32, 33].

Subjects were divided into four categories: (1) no knee pain or radiographic OA (Pain-/ROA-); (2) radiographic OA with no pain (ROA); (3) knee pain with no radiographic OA (POA); (4) pain and radiographic OA (PROA). Person-level OA was calculated by assessing the OA status for each joint and using the ‘highest’ level of OA based on this system. For example, if a subject had no knee pain or radiographic OA (cat 1) in their right knee and radiographic OA with no pain (cat 2) in their left knee, their person-level knee OA status would be radiographic OA with no pain (cat 2).

### Primary outcome: time-to-mortality

Each cohort contained a status variable (dead/alive) and a time-to-censoring variable for each participant. Three cohorts (Chingford, Johnston County, TasOAC) determined the date of death using nationally linked records, while the remaining cohorts used other methods to determine the date of death such as updates from Primary Care systems, death registries or municipal administration, family, medical records, and periodic examinations or contacts.

In cohorts where subjects were lost to follow-up at an unknown date, the previous visit when subjects had data was used as the last date where mortality status was known. Time-to-status was calculated from the baseline visit, determined by when knee X-rays and pain were assessed, to the last date that the subject’s status was known. Survival was calculated using person-years attributing to the analysis.

### Potential confounders

The potential confounders accounted for in this analysis were: age; sex; race; BMI; smoking; drinking; cardiovascular disease (CVD); and diabetes. These were based on clinical applicability and consistent availability across each cohort. To be modelled consistently between cohorts, variables were categorised into the broadest level of information available in any single cohort. For example, one cohort contained detailed data on the lifetime use of all tobacco products enabling the generation of a ‘dose’, while another cohort simply asked whether they were current, former, or never smokers. This second option was then generated for each cohort. Pain medication, such as NSAIDs, was not considered a potential confounder in this analysis, as it is on the causal pathway between painful OA and mortality, and a mediation analysis on this scale would not have been feasible due to both limitations in the data and in the methodology.

*Age* was defined as age at the time of baseline clinic visit when OA variables were assessed. *Race* was included as a potential confounder for any cohort which had more than one race category. Chingford, TasOAC, and Framingham have predominantly Caucasian participants; Johnston County and MOST have both Caucasian and African American subjects; and Wuchuan has predominantly Chinese subjects. *BMI* was calculated for each cohort using height and weight variables (weight/height in metres [2]). Extreme values were identified in several cohorts; however, due to the wide variety of subjects found in our dataset, we only excluded impossible (i.e. outside any known values) rather than improbable values. Smoking, Alcohol, Diabetes, and CVD were all generated as binary variables. *Smoking* was calculated with current/former smokers and never smokers. *Alcohol* was grouped by more than one drink per week versus none or one drink per week. *Diabetes* was based on the presence of self-reported clinically diagnosed diabetes, and *CVD* was calculated using self-reported responses to previous ischaemic heart disease, and general heart problems.

### Statistical methods: descriptive statistics

Descriptive statistics [percentages, means (standard deviations), and medians (inter-quartile ranges)] were calculated for baseline characteristics of all cohorts using all available data. The difference between baseline characteristics in subjects with and without complete data (OA and confounders) was calculated using t tests (or Wilcoxon–Mann–Whitney) for continuous variables and Chi-square tests (or Fisher’s exact) for binary and categorical variables. Descriptive statistics for baseline characteristics and time-to-mortality data were stratified by the categories, no pain/no ROA, POA, ROA and PROA.

### Statistical methods: missing data

There were three potential types of missing data to consider within our analyses. To identify data that was missing at random (MAR) and missing completely at random (MCAR), we tested patterns and predictors of missingness for all exposures and potential confounders. We identified several MAR variables and ensured to include any required predictors in the imputation model. All other variables were assumed to be MCAR, a non-testable assumption. There were also systematically missing variables, which were missing in their entirety in a single cohort. Appendix 2 shows the systematically missing and MAR/MCAR variables for each cohort.

Multiple imputation with chained equations (MICE) was used to impute any missing data for both the primary risk factor and for confounders [34, 35]. Systematically missing variables (i.e., variables which were missing in their entirety) were excluded from all models and analyses. Participants with missing mortality data were excluded from all analysis (cohorts had no more than three percent missing mortality data). The Nelsen–Aalen estimator was used to approximate the baseline hazards in the imputation models [36]. Variables used for the imputation models were congruent with the analysis model described in the next section. Missing PROA and race were modelled using multinomial logistic regression; BMI by linear regression; sex, smoking, alcohol, CVD, and diabetes by logistic regression. Age was modelled by predictive mean matching due to non-normality from being restricted between ages 45 and 80 [37].

### Statistical methods: survival analysis

Cox proportional hazard regression models were used to estimate hazard ratios (HRs) and 95% confidence intervals (95% CIs) between three OA categories (POA, ROA, PROA) and the time-to all-cause-mortality using no pain/no ROA as the comparator group for each analysis. Three models were run: (1) univariable models assessed OA alone; (2) adjusted for age, sex and race; (3) adjusted for age, sex, race, BMI, smoking, alcohol, CVD, and diabetes. Models run in the Johnston County cohort also included a variable for recruitment wave. Several cohorts were systematically missing key potential confounders (primarily smoking and alcohol) (Appendix 2).

To satisfy the assumptions of the Cox proportional hazard model linearity was assessed between continuous variables (age and BMI) and time to death using fractional polynomials and kernel. The proportional hazard assumption of the primary risk factor (OA) was tested using Schoenfeld residuals. Due to the violation of this proportionality assumption, Johnston County was truncated to the 13-year follow-up post hoc, which was the maximum follow-up time of one of the recruitment waves. This corrected the violation of proportionality for the PROA variable, although reduced the power of this cohort. A priori interactions of OA and age, and OA and BMI were tested in all cohorts.

### Statistical methods: individual participant data analysis

Individual participant-level meta-analysis methods were utilised, using a two-staged approach [21, 38]. In the first stage, hazard ratios (HR) and 95% confidence intervals (CI) were first produced for each individual cohort. Data were pooled in the second stage using random-effects analysis, using the Hartung–Knapp estimation to account for uncertainty around the tau statistic [39, 40].

The Stata *admetan* command was used to produce the pooled estimates in addition to forest plots which graphically demonstrate the results [41]. All analyses were conducted using Stata version 13·0 statistical software (StataCorp, College Station, Texas, USA).

### Role of funding source

**Fig. 1:**
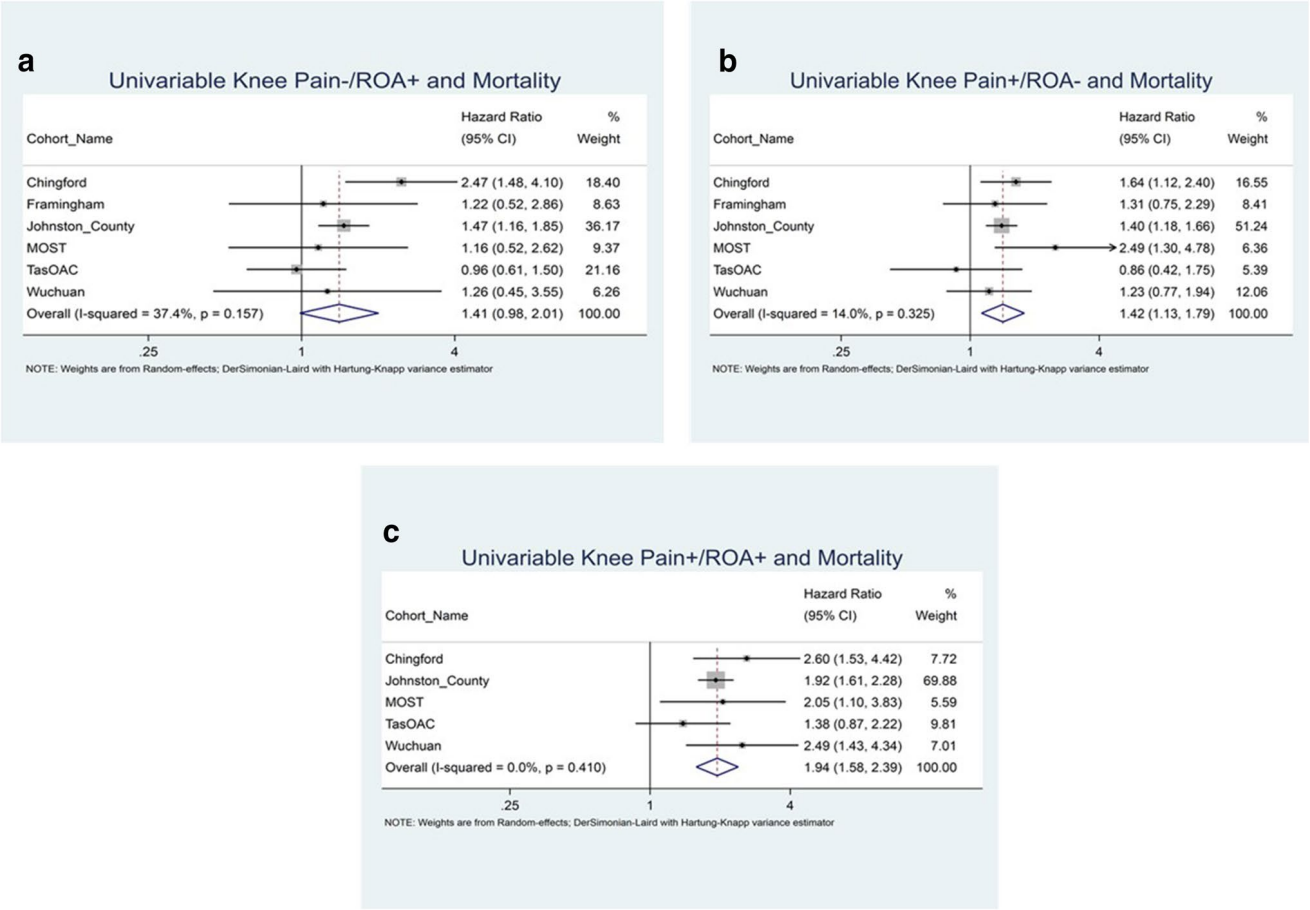
**a**–**c** Forest plots of univariable models: **a** ROA; **b** POA; **c** PROA compared to Pain-/ROA-

Versus Arthritis UK (formally Arthritis Research) had no role in study design, data collection, data analysis, data interpretation, or writing of the report. Members of the PCCOA steering committee from Osteoarthritis Research Society International (a non-profit scientific organisation) had roles in study development and interpretation as outlined in the author contribution section with all contributors named in the writing group. The corresponding author had full access to all the data in the study and had final responsibility for the decision to submit for publication (Fig. 1).

## Results

10,723 participants in six cohorts from four countries were included in the analyses. All cohorts had less than 3% missing mortality data and less than 12% missing risk factor or confounder data. Participants with missing mortality data were excluded, whilst those missing risk and confounder data were included in imputed analyses. In several cohorts, there was a statistically significant difference in OA, age, BMI, diabetes, and CVD in subjects with and without missing data (Appendix 3).

Table 1 shows the baseline demographics for all cohorts stratified by baseline OA. Median follow-up for this analysis ranged from 5·6 to 20·0 years after baseline. There was substantial variability in the baseline age (54.3–62.7 years), BMI (22.5–30.7 kg/m^2^), prevalence of PROA (6.7–33.3%), and the duration of follow-up in each cohort, such that the percentage of subjects that died in each cohort ranged from 2.9 to 22.3% (Table 2).

**Table 1:** Cohort (1–3) baseline demographics for all subjects and stratified by baseline knee OA status

Baseline demographics	Chingford					Johnston County					Framingham				
	None	ROA	POA	PROA	All	None	ROA	POA	PROA	All	None	ROA	POA	PROA	All
*N*	588 (61.3%)	75 (7.8%)	232 (24.2%)	64 (6.7%)	992	1707 (45.4%)	378 (10.1%)	1023 (27.2%)	654 (17.4%)	3918	594 (67.0%)	63 (7.1%)	181 (20.4%)	48 (5.4%)	905
Age	53.8 (5.9)	57.2 (5.8)	53.9 (5.9)	57.1 (5.4)	54.3 (6.0)	58.3 (9.1)	62.8 (9.4)	58.8 (9.1)	63.5 (9.3)	60.0 (9.5)	55.6 (7.5)	57.1 (7.5)	56.0 (7.7)	59.8 (7.2)	56.0 (7.6)
Sex (female)	588 (100%)	75 (100%)	232 (100%)	64 (100%)	992 (100%)	1018 (59.6%)	228 (60.3%)	662 (64.7%)	440 (67.3%)	2457 (62.7%)	297 (50.0%)	30 (47.6%)	108 (59.7%)	26 (54.2%)	474 (52.5%)
Race															
Caucasian	..	..	..	..	..	1168 (68.4%)	251 (66.4%)	648 (63.3%)	399 (61.0%)	2568 (65.5%)	594 (100%)	63 (100%)	181 (100%)	48 (100%)	905 (100%)
African American	..	..	..	..	..	539 (31.6%)	127 (33.6%)	375 (36.7%)	255 (39.0%)	1352 (34.5%)	..	..	..	..	..
Chinese	..	..	..	..	..	..	..	..	..	..	..	..	..	..	..
Other	..	..	..	..	..	..	..	..	..	..	..	..	..	..	..
BMI	24.9 (3.9)	27.3 (4.8)	25.9 (4.3)	28.8 (5.2)	25.6 (4.3)	27.9 (5.0)	30.2 (6.7)	30.0 (6.1)	33.6 (8.0)	29.7 (6.4)	26.7 (4.2)	28.6 (5.5)	27.9 (4.9)	30.6 (5.9)	27.3 (4.6)
Alcohol (one or more)	237 (40.3%)	21 (28.0%)	81 (34.9%)	21 (32.8%)	375 (37.8%)	..	..	..	..	..	417 (70.4%)	43 (68.3%)	116 (64.1%)	36 (75.0%)	623 (69.1%)
Smoking (ex/current)	267 (45.4%)	39 (52.0%)	107 (46.1%)	29 (45.3%)	458 (46.2%)	870 (52.0%)	162 (43.3%)	566 (56.8%)	287 (44.7%)	1933 (50.9%)	388 (65.4%)	35 (55.6%)	121 (66.9%)	24 (50.0%)	580 (64.2%)
CVD (yes)	19 (3.2%)	3 (4.1%)	6 (2.6%)	2 (3.1%)	31 (3.1%)	413 (24.2%)	90 (23.8%)	356 (34.8%)	198 (30.3%)	1098 (28.0%)	19 (3.3%)	0 (0.0%)	10 (5.5%)	1 (2.1%)	30 (3.4%)
Diabetes (yes)	5 (0.9%)	0 (0.0%)	4 (1.7%)	0 (0.0%)	9 (0.9%)	162 (9.5%)	45 (11.9%)	152 (14.9%)	128 (19.6%)	509 (13.0%)	21 (3.6%)	1 (1.6%)	13 (7.2%)	4 (8.5%)	40 (4.4%)
*N*	827 (28.5%)	503 (17.3%)	608 (20.9%)	968 (33.3%)	2936	206 (23.4%)	372 (42.3%)	83 (9.4%)	219 (24.9%)	955	469 (46.2%)	42 (4.1%)	398 (39.2%)	107 (10.5%)	1017
Age	61.2 (8.0)	64.5 (8.0)	60.4 (8.0)	63.7 (7.9)	62.5 (8.1)	62.0 (7.3)	63.2 (7.5)	60.5 (6.3)	63.3 (7.4)	62.7 (7.4)	55.8 (7.3)	62.2 (8.9)	55.1 (7.1)	61.4 (8.5)	56.4 (7.7)
Sex (female)	448 (54.2%)	289 (57.5%)	390 (64.1%)	632 (65.3%)	1775 (60.5%)	96 (46.6%)	189 (50.8%)	34 (41.0%)	119 (54.3%)	477 (50.0%)	203 (43.3%)	26 (61.9%)	212 (53.3%)	74 (69.2%)	516 (50.7%)
Race															
Caucasian	734 (88.8%)	436 (86.7%)	498 (81.9%)	781 (80.1%)	2470 (84.1%)	..	..	..	..	..	..	..	..	..	..
African American	82 (9.9%)	61 (12.1%)	96 (15.8%)	179 (18.5%)	426 (14.5%)	..	..	..	..	..	..	..	..	..	..
Chinese	..	..	..	..	..	..	..	..	..	..	469 (100%)	42 (100%)	398 (100%)	107 (100%)	1017 (100%)
Other	11 (1.3%)	6 (1.2%)	14 (2.3%)	8 (0.8%)	40 (1.3%)	..	..	..	..	..	..	..	..	..	..
BMI	29.0 (4.6)	30.6 (5.4)	29.5 (5.4)	32.8 (6.8)	30.7 (5.9)	26.9 (4.3)	27.3 (4.3)	28.1 (4.8)	29.5 (5.5)	27.8 (4.7)	21.9 (3.1)	23.3 (3.4)	22.6 (3.1)	24.0 (4.0)	22.5 (3.3)
Alcohol (one or more)	..	..	..	..	..	109 (52.9%)	189 (50.8%)	44 (53.0%)	101 (46.1%)	476 (49.8%)	..	..	..	..	..
Smoking (ex/current)	380 (46.0%)	213 (42.4%)	261 (42.9%)	438 (45.3%)	1305 (44.5%)	104 (50.5%)	185 (49.9%)	51 (61.5%)	109 (49.8%)	480 (50.5%)	..	..	..	..	..
CVD (yes)	76 (9.4%)	62 (12.6%)	68 (11.5%)	129 (13.8%)	339 (11.9%)	14 (7.1%)	30 (8.5%)	5 (6.3%)	18 (8.5%)	74 (8.1%)	47 (10.0%)	2 (4.8%)	51 (12.8%)	19 (17.8%)	120 (11.8%)
Diabetes (yes)	59 (7.3%)	43 (8.6%)	68 (11.4%)	134 (14.4%)	307 (10.7%)	13 (6.6%)	15 (4.2%)	6 (7.5%)	15 (7.1%)	54 (5.59%)	2 (0.4%)	0 (0.0%)	2 (0.5%)	1 (0.9%)	5 (0.45%)

**Table 2:** Mortality information by baseline knee osteoarthritis status

Mortality information	Osteoarthritis			
	None	ROA	POA	PROA
Chingford				
Total *N*	588	75	232	64
No of deaths	67	19	42	17
Median follow-up	20.0 (20.0, 20.0)	20.0 (19.4, 20.0)	20.0 (20.0, 20.0)	20.0 (18.6, 20.0)
Median time-to-death	14.2 (8.4, 17.1)	13.5 (8.0, 16.2)	14.1 (10.6, 17.9)	12.1 (6.7, 16.2)
Johnston County				
Total *N*	1707	378	1023	654
No of deaths	300	96	250	208
Median follow-up	12.5 (9.4, 13.0)	12.0 (7.5, 13.0)	11.5 (9.3, 13.0)	10.8 (8.3, 13.0)
Median time-to-death	7.3 (4.2, 10.6)	7.4 (4.3, 10.6)	7.1 (4.3, 9.9)	6.7 (3.7, 9.8)
Framingham				
Total *N*	594	63	181	48
No of deaths	44	6	17	1
Median follow-up	11.8 (10.8, 12.5)	12.3 (11.53, 12.7)	11.6 (10.8, 12.5)	12.2 (11.8, 12.7)
Median time-to-death	8.3 (4.9, 10.7)	6.0 (3.7, 8.3)	9.0 (7.2, 10.6)	13.2 (13.2, 13.2)
MOST				
Total *N*	827	503	608	968
No of deaths	14	10	26	34
Median follow-up	5.6 (5.5. 5.8)	5.6 (5.5. 5.8)	5.6 (5.5. 5.8)	5.6 (5.5. 5.8)
Median time-to-death	2.7 (1.9, 4.8)	4.8 (4.2, 5.3)	4.0 (2.7, 4.9)	3.2 (1.8, 5.0)
TasOAC				
Total *N*	206	372	83	219
No of deaths	31	53	10	39
Median follow-up	12.0 (8.8, 13.1)	11.9 (8.2, 12.9)	11.6 (9.7, 13.0)	10.4 (5.0, 12.6)
Median time-to-death	9.1 (6.4, 10.9)	7.9 (6.1, 10.5)	9.8 (6.4, 10.8)	6.4 (3.7, 9.5)
Wuchuan				
Total *N*	469	42	698	107
No of deaths	36	4	37	19
Median follow-up	8.3 (8.3, 8.3)	8.3 (8.3, 8.3)	8.3 (8.3, 8.3)	8.3 (8.3, 8.3)
Median time-to-death	5.9 (4.0, 7.5)	5.4 (3.3, 6.4)	5.7 (3.8, 6.8)	4.8 (3.9, 5.9)

**Fig. 2:**
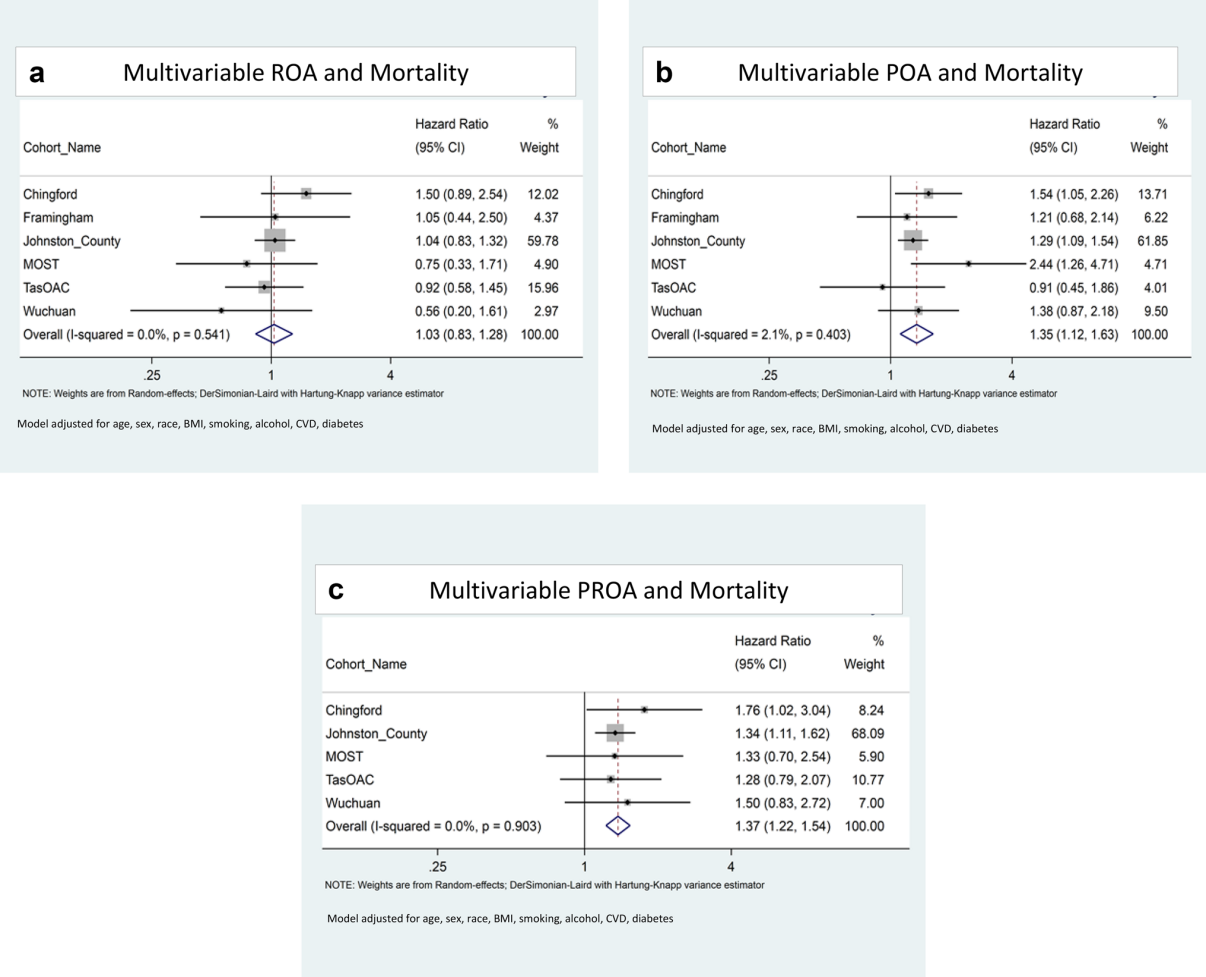
**a**–**c** Forest plots of fully adjusted models: **a** ROA; **b** POA; **c** PROA compared to Pain-/ROA-

The univariable meta-analysis (Fig. 2) shows a non-significant pooled hazard ratio (HR and 95% confidence interval) of 1.41 (0.98, 2.01) for ROA. Both POA and PROA were significantly associated with reduced time-to-mortality (1.42 [1.13, 1.79], and 1.94 [1.58, 2.39], respectively) when compared with participants with no pain or ROA. In the model adjusted for age, sex, and race only, the effect size was attenuated and remained non-significant for ROA (1.0 [0.70, 1.44]); increased slightly and remained significant for POA (1·44 [1.11, 1.85]); and was attenuated for PROA (1.36 [1.18, 1.56]) compared with the univariable models.

In the fully adjusted model (age, sex, race, BMI, smoking, alcohol consumption, CVD, and diabetes), ROA remained non-significant, and participants with POA or PROA had a 35% (HR 1·35 [1.13, 1.63]) and 37% (HR 1·37 [1.22, 1.54]) increased association with reduced time-to-mortality, respectively (Fig. 2).

## Discussion

### Key results

This individual participant-level meta-analysis of over 100,000 people from four countries revealed that participants with knee pain only, or a combination of knee pain and radiographic OA, had an increased association with reduced time-to-mortality, independent of age, sex, and race (HRs of 1.36–1.44). To explore whether the association could be explained by co-morbid conditions, the models were further adjusted for BMI, smoking, alcohol, CVD, and diabetes. The results remained consistent with HRs of 1.35 for those with POA and 1.37 for those with PROA, compared to participants without knee pain and ROA (pain-/ROA-). Interestingly, we did not observe an association with time-to-mortality in participants with radiographic changes alone (ROA), suggesting that it is pain or some functional consequence of pain such as walking disability or reduced physical activity, rather than the structural aspect of knee OA, that may be driving the increased association with premature mortality [42, 43]. While many studies have found an association between OA-related pain and premature mortality, the potential pathways that explain this association is still unclear. A study using large population-based data sets to investigate the effect of pain phenotype on the association between pain and mortality found that the impact of pain in daily life was more important than the presence or extent of pain in the relationship between pain and mortality [44]. Findings from one of the same cohorts examining the potential mechanisms between OA and all-cause mortality highlighted frequent walking as a potential target to reduce all-cause mortality. While anxiety, depression, and unrefreshed sleep had statistically significant effects, the extent of their mediation effect had low clinical significance [45].

### Results in the context of other studies

Three recent meta-analyses found no association between OA and mortality, with pooled effect sizes of 0.91 (0.68, 1.23), 1.06 (0.88, 1.28), and 1.21 (0.82, 1.78) in a knee only analysis [7–9]. All three articles combined results of studies which used multiple forms of OA diagnosis, including clinician diagnosed OA, self-reported clinical diagnosis, pain, and radiographic OA, increasing the measurement error of the OA variable. Two of the meta-analyses combined studies with knee, hip, and hand data into a single effect size [7, 9]. Individual studies, which assessed knee pain and radiographic OA with mortality, tend to report higher effect sizes more consistent without our results. For instance, Liu et al. [15] reported a borderline significant HR of 1.90 (1.00, 3.50), while Tsuboi et al. [12] reported a significant HR of 2.32 (1.41, 3.80), as did Kluzek et al. [14], HR of 1·47 (1.08, 2.01). Cleveland et al. observed an increased risk of all-cause mortality in participants with knee pain alone (HR of 1.19 [1.04–1.35]) and those with symptomatic knee OA (HR of 1.17 [1.03–1.34]) [46]. Castano Betancourt et al. and Neusch et al. combined hip and knee pain/ROA, and both found a significant association with premature mortality (1.23 and 1.55, respectively) [11, 47], supporting the concept that the association of knee OA with reduced time-to-mortality may be driven by pain rather than by structural changes identified by radiographs. In this analysis, we treated a number of comorbidities as potential confounders; however, the relationship between these comorbidities and OA is poorly understood and may ultimately be part of the causal pathway; therefore, the associations we found here may not represent a causal association between OA and mortality. We could be underestimating this association if some of the potential confounders are actually mediators on the causal pathway, and we may be overestimating the association depending on how well our adjusted models are accounting for confounding.

Patients with OA have on average 2·6 moderate-to-severe co-morbidities [48] and 31% of patients have five or more other chronic conditions [49]. Our fully adjusted models, which included lifestyle factors and cardiovascular conditions, did not change substantially from the models adjusted for age, sex, and race. This may indicate that the additional potential confounders we have adjusted for do not have a substantial confounding effect on the association between OA and reduced time-to-mortality. This suggests that either the association is driven by OA or is due to residual confounding caused by measurement error in self-reported variables and/or by the lack of potential confounders such as physical activity and occupation. An additional potential source of unmeasured confounding is the pain sensitization, which may effect the relation of painful knee OA and mortality, and should be pursued in future research. The current study focuses on the knee; however, it is known that limitations in activities of daily living and mobility vary according to hip or knee site [50]; previous studies have also found an increased risk of mortality in individuals with hip symptoms [51].

### Strengths and limitations

A limitation of this study is that the included cohorts were designed as independent studies and were not originally designed to be directly compared to one another. Therefore, osteoarthritis was assessed differently between cohorts. It is known that even small variations in the way a pain question is worded, or X-rays are graded, can result in differences in OA prevalence [22, 52]. To minimise this variation, we made every effort to harmonise pain and ROA variables between cohorts by conducting an international expert consensus study [22].

One of the strengths of our study, unlike traditional meta-analyses, is that we actively sought cohorts that had not previously published on the association between OA and mortality, to avoid publication bias. To also capture people without the symptomatic aspects of OA, we restricted our studies to those that included the general population and one enhanced risk factor cohort. These people would not be included in clinical OA cohorts, which is a known issue in the accurate reporting of the true burden of OA [53, 54].

The MOST cohort included additional focussed recruitment to include a larger proportion of participants that were older, female, overweight, or had knee surgery/injury, all factors associated with an increased risk of OA. Therefore, the reference group (without pain or ROA) is likely to have a higher prevalence of OA risk factors than the pain and ROA-free group in other cohorts, which may have biased our results toward the null in this cohort.

The follow-up of the included studies ranged from 5.6 to 20.0 years; however, only baseline knee OA and confounders were included in the analysis, meaning that participants may have changed OA categories after the baseline visit, resulting in possible misclassification bias. A further potential limitation is that the age of our participants at baseline ranged between 45 and 80; however, the mean age between cohorts was relatively similar with lowest having a mean age of 56.0 and the highest with a mean age of 64.4 (Table 1).

Both a strength and limitation of the current study is that we included cohorts from different countries, with different races, cultures and health care systems. Confounders were harmonised using the least detailed information available in any single cohort at the baseline visit only, which likely increased our risk of residual confounding in our models. However, by harmonising the individual confounders and adjusting for them consistently between studies, we have reduced unnecessary heterogeneity between studies. Therefore, remaining differences between cohorts are more likely to reflect racial, country, and/or cultural variations rather than how variables were defined, or which statistical models were used.

Previous individual cohort or meta-analysis studies have suggested that a large proportion of the increased risk of mortality is due to cardiovascular mortality [9, 14]. Cause-specific mortality was not available in the majority of our cohorts and justifies further investigation. Likewise, medical detail was not available across all cohorts to consider the effect of pain-relieving medications. Pain medication is on the casual pathway between painful OA and mortality, and by not including it in our model, our associations are ultimately combining both the direct effect of OA on mortality and the indirect effect of OA through pain medication on mortality. Future research using mediation analysis will help to clarify this pathway.

IPD meta-analyses are time-consuming and resource intensive compared with traditional meta-analyses; however, they allow for standardising exposures, outcomes, and statistical methods, and, more importantly, avoid publication bias by not being limited to the inclusion of previously published studies, which is rarely done in the traditional meta-analysis.

## Conclusion

This study is the first individual participant-level data meta-analysis of knee osteoarthritis and premature mortality. It demonstrates that participants with knee pain only or a combination of knee pain and radiographic OA had a 35–37% increased association with reduced time-to all-cause-mortality independent of age, sex, race, BMI, smoking, alcohol, CVD, or diabetes. With the increasing prevalence of knee OA, it is essential that clinicians and public health bodies are aware of the potential that people with OA may have an increased burden of premature mortality compared to people without OA. This finding highlights that osteoarthritis is a serious disease and supports the need for further research to identify whether OA-related mechanisms are causally associated with premature mortality.

## Appendix 2: Cohort inclusion/exclusion criteria and missing data

**Table:**

	Chingford	Johnston County	Framingham	MOST	TasOAC	Wuchuan
Original cohort *N*	1003	4197	1166	3026	1100	1030
Subjects meeting inclusion criteria						
Without Rheumatoid Arthritis	996	..	1154	2938	983	..
Age (45–80 years)	992	3968	905	2938	980	1017
Mortality data	992	3918	905	2936	955	1017
Total Subjects meeting Inclusion Criteria*	992	3918	905	2936	955	1017
Subjects without missing data						
PROA	959	3762	905	2906	880	1016
Sex	992	3762	886	2906	880	1016
Race	992	3762	886	2906	..	1016
BMI	959	3756	886	2905	880	1016
Alcohol	959	..	884	..	880	..
Smoking	959	3681	883	2905	879	..
CVD	956	3681	869	2824	843	1016
Diabetes	956	3676	865	2762	843	1016
Total subjects without missing data	956	3676	865	2762	843	1016

^a^Total *N* after imputation

## Appendix 3: Complete case vs subjects with missing values for each cohort

See Tables 3, 4, 5, 6, 7, 8.

**Table 3:** Johnston County complete case vs subjects with any missing values for risk factor and confounders

Baseline variable	Complete case	Missing values	*p* value
*N* = 3918	3762	156	
Osteoarthritis			
None	1707 (45.4%)	0	
ROA	378 (10.1%)	0	
POA	1023 (27.2%)	0	
PROA	654 (17.4%)	0	
Age	59.8 (9.4)	63.3 (10.4)	0.000
Sex (% female)	2348 (62.4%)	109 (69.9%)	0.059
Race			
Caucasian	2466 (65.6%)	102 (65.4%)	0.966
African American	1296 (34.5%)	54 (34.6%)	
BMI (continuous)	29.7 (6.4)	29.3 (6.1)	0.4253
Ex/current smoking (binary)	1885 (51.1%)	48 (44.0%)	0.145
CVD2 (heart/stroke)	1057 (28.1%)	41 (26.3%)	0.621
Diabetes	487 (13.0%)	22 (14.5%)	0.588

*t* tests (or Wilcoxon–Mann–Whitney test) for continuous variables and Chi^2^-tests (or Fishers exact) for categorical variable

**Table 4:** Framingham complete case vs subjects with any missing values for risk factor and confounders

Baseline variable	Complete case	Missing values	*p* value
*N* = 905	886	19	
Osteoarthritis			
None	594 (67.0%)	0	
ROA	63 (7.1%)	0	
POA	181 (20.4%)	0	
PROA	48 (5.4%)	0	
Age	56.0 (7.6)	57.2 (7.6)	0.466
Sex (% female)	461 (52.0%)	13 (68.4%)	0.157
Race			
Caucasian	886 (100%)	19 (100%)	
BMI (continuous)	27.3 (4.6)	26.8 (3.1)	0.809
Ex/current Smoking (binary)	568 (64.2%)	12 (63.2%)	0.927
CVD2 (heart/stroke)	30 (3.4%)	0	
Diabetes	39 (4.4%)	1 (5.3%)	0.860

*t* tests (or Wilcoxon–Mann–Whitney test) for continuous variables and Chi^2^ tests (or Fishers exact) for categorical variables

**Table 5:** Chingford complete case vs subjects with any missing values for risk factor and confounders

Baseline variable	Complete case	Missing values	*p* value
*N* = 857	683	174	
Osteoarthritis			
None	483 (70.7%)	0	
ROA	129 (18.9%)	0	
POA	41 (6.0%)	0	
PROA	30 (4.4%)	0	
Age	57.9 (6.0)	58.1 (5.9)	0.697
BMI (continuous)	26.3 (4.4)	26.4 (4.3)	0.735
Ex/current smoking (binary)	317 (46.4%)	70 (40.2)	0.143
CVD2 (heart/stroke)	25 (4.3%)	10 (7.4%)	0.134
Diabetes	6 (0.9%)	3 (1.7%)	0.329

*t* tests (or Wilcoxon–Mann–Whitney test) for continuous variables and Chi^2^-tests (or Fishers exact) for categorical variables

**Table 6:** MOST complete case vs subjects with any missing values for risk factor and confounders

Baseline variable	Complete case	Missing values	*p* value
*N* = 2936	2906	30	
Osteoarthritis			
None	827 (28.5%)	0	
ROA	503 (17.3%)	0	
POA	608 (20.9%)	0	
PROA	968 (33.3%)	0	
Age	62.5 (8.1)	64.3 (6.9)	0.215
Sex (% female)	1759 (60.5%)	16 (53.3%)	0.423
Race			
Caucasian	2449 (84.3%)	21 (70.0%)	0.058
African American	418 (14.4%)	8 (26.7%)	
Other	39 (1.3%)	1 (3.3%)	
BMI (continuous)	30.7 (5.9)	30.4 (6.7)	0.805
Ex/current Smoking (binary)	1292 (44.5%)	13 (43.3%)	0.902
CVD2 (heart/stroke)	335 (11.9%)	4 (13.8%)	0.749
Diabetes	304 (10.7%)	3 (10.0%)	0.901

*t* tests (or Wilcoxon–Mann–Whitney test) for continuous variables and Chi^2^-tests (or Fishers exact) for categorical variables

**Table 7:** TaSOAC complete case vs subjects with any missing values for risk factor and confounders

Baseline variable	Complete case	Missing values	*p* value
*N* = 445	410	35	
Osteoarthritis (no-side specific pain)			
None	96 (23.4%)	0	
ROA	157 (38.3%)	0	
POA	42 (10.2%)	0	
PROA	115 (28.1%)	0	
Age	64.4 (7.9)	65.1 (8.7)	0.6464
Sex (% female)	209 (51.0%)	18 (51.4%)	0.959
Race			
Caucasian white	263 (98.1%)	19 (100%)	0.835
Asian	2 (0.8%)		
Indigenous Australian	3 (1.1%)		
BMI (continuous)	28.2 (5.2)	27.6 (4.0)	0.4707
Ex/current Smoking (binary)	224 (54.6%)	20 (58.8%)	0.637
CVD2 (heart/stroke)	44 (11.7%)	5 (17.2%)	0.378
Diabetes	36 (9.6%)	4 (13.8%)	0.463

*t* tests (or Wilcoxon–Mann–Whitney test) for continuous variables and Chi^2^-tests (or Fishers exact) for categorical variables

**Table 8:** Wuchuan complete case vs subjects with any missing values for risk factor and confounders

Baseline variable	Complete case	Missing values	*p* value
*N* = 1017	1016	1	
Osteoarthritis			
None	469 (46.2%)		
ROA	42 (4.1%)		
POA	398 (39.2%)		
PRO	107 (10.5%)		
Age	56.4 (7.7)		
Sex (% female)	515 (50.7%)		
BMI [mean (SD)]	22.5 (3.3)		
CVD (heart/stroke) (%)	119 (11.7%)		
Diabetes	5 (0.5%)		

Data is not presented for missing values, and no tests were done comparing groups due to small number of participants with missing data
